# Quantum biophysics of photosynthesis: evolutionary tuning for robust performance under natural light

**DOI:** 10.3389/fpls.2026.1881848

**Published:** 2026-06-30

**Authors:** Adriana M. De Mendoza

**Affiliations:** Physics Department, Pontificia Universidad Javeriana, Bogota, Colombia

**Keywords:** natural illumination, photoprotection, photosynthetic adaptation, quantum photosynthesis, robustness

## Abstract

Photosynthesis converts sunlight into chemical energy through networks of pigment–protein complexes that operate reliably under warm, noisy, and highly variable environmental conditions. Over the past decades, advances in spectroscopy and physically grounded modeling have shown that excitation energy transfer in these systems is governed by quantum-mechanical interactions shaped by environmental fluctuations. However, understanding photosynthetic function requires placing these microscopic processes within the broader context of natural illumination, where incoherent sunlight and multiple interacting timescales drive photosynthetic systems toward dynamically stabilized operating regimes. Under these conditions, ultrafast excitonic and vibronic dynamics remain relevant at short spatial and temporal scales, while overall photosynthetic behavior emerges from the interplay between energy transfer, dissipation, photoprotection, and adaptation under fluctuating environmental conditions. Building on this physical picture, this Perspective discusses photosynthetic function as a robust energy-management process shaped by evolutionary adaptation to natural illumination. Across bacteria, cyanobacteria, algae, and plants, photosynthetic systems operate under common physical constraints, including the stochastic nature of sunlight, finite excitation-transfer and charge-separation rates, thermal fluctuations, and the need to avoid photodamage under variable irradiance. These constraints influence the temporal organization of excitation transport, dissipation, and photoprotective responses. In this view, photosynthetic function depends not only on efficient excitation transfer, but also on maintaining stable and adaptable energy flow across changing environmental conditions. By integrating quantum physics, biological regulation, and ecological variability, this interdisciplinary perspective highlights how physical constraints shape robust photosynthetic function under realistic environmental conditions.

## 1 Introduction

Understanding photosynthesis requires an interdisciplinary perspective integrating physics, chemistry, and biology to explain how living systems achieve reliable energy conversion under fluctuating environmental conditions. At its core, photosynthesis involves the capture and redistribution of electronic excitation energy across pigment–protein complexes toward reaction centers, where charge separation initiates the conversion of light into chemical potential. This process operates robustly despite warm, noisy, and dynamically fluctuating biological environments, making photosynthetic systems paradigmatic examples of robust energy conversion in complex living systems Blankenship (2014); Scholes et al. (2011). Over the past two decades, ultrafast spectroscopy and theoretical modeling have shown that excitation energy transfer in photosynthesis can be described within the framework of open quantum systems.

**Figure 1:**
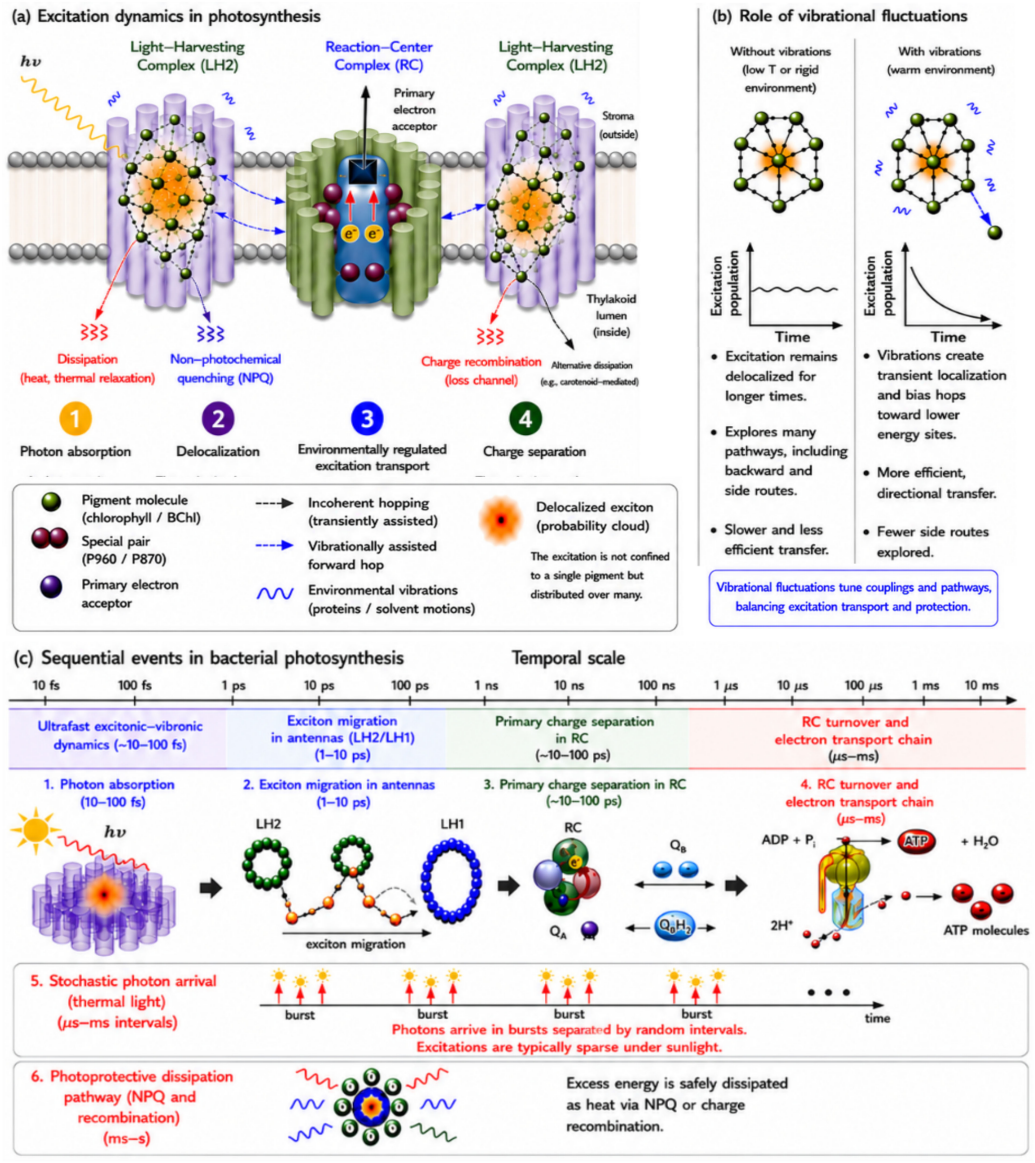
Excitation dynamics and multiscale regulation in photosynthetic systems. **(a)** Excitation transport from light-harvesting complexes to the reaction center (RC), regulated by vibrational interactions, environmental fluctuations, and dissipative pathways. **(b)** Environmental and vibronic interactions dynamically modulate excitation redistribution and dissipation under physiological conditions. **(c)** Multiscale organization of photosynthetic processes in purple bacteria, from ultrafast excitonic–vibronic dynamics to slower RC turnover and photoprotective regulation under stochastic natural illumination. Transient ultrafast coherent signatures should not be interpreted as evidence of persistent long-lived electronic coherence under physiological sunlight conditions (Cao et al., 2020).

The electronic excitation of very close individual pigments gives rise to partially delocalized excitonic states extending across multiple chromophores, whose dynamics are strongly influenced by interactions with the surrounding protein environment. Environmental fluctuations, molecular vibrations, and dissipative interactions continuously shape excitation redistribution and relaxation dynamics, motivating theoretical descriptions based on environment-assisted quantum transport (ENAQT) (Plenio and Huelga, 2008; Rebentrost et al., 2009b, Rebentrost et al., 2009a; Chin et al., 2013; Huelga and Plenio, 2013; Ishizaki and Fleming, 2009; Olaya-Castro et al., 2008). More recent theoretical and experimental work, however, has revised the interpretation of coherent phenomena in photosynthetic systems, showing that many long-lived oscillatory signals observed in ultrafast spectroscopic experiments arise predominantly from mixed excitonic–vibrational (vibronic) dynamics rather than from persistent purely electronic coherence (Cao et al., 2020; Jumper et al., 2018; Ruan and Weng, 2026; Jha et al., 2026; Lim et al., 2015; Arsenault et al., 2021; Harush and Dubi, 2021). Within this modern perspective, excitation transport emerges from the interplay between transient excitonic delocalization, vibronic interactions, thermal fluctuations, and environmentally regulated dissipation under physiological conditions. A schematic representation of excitation delocalization, environmentally assisted transport, vibronic interactions, and reaction-center transfer is shown in **Figures 1a, b**.

Natural photosynthesis operates under sunlight, a broadband thermal radiation field fundamentally different from the coherent laser sources commonly used in laboratory experiments. Sunlight exhibits stochastic intensity fluctuations and finite temporal correlations that influence the timing and distribution of excitation events in the photosynthetic apparatus (Mandel and Wolf, 1995; Brumer, 2018; Mančal and Valkunas, 2010; De Mendoza et al., 2020, De Mendoza et al., 2017). Under such conditions, excitation dynamics are additionally shaped by pigment coupling, environmental fluctuations, and finite reaction-center turnover times (Fassioli et al., 2014; Manrique et al., 2016). Excitation transport therefore spans timescales ranging from ultrafast excitonic motion (fs-ps) to slower photoprotective and biochemical responses (*µ*s-ms) as depicted in **Figure 1c** (Blankenship, 2014; Ruban, 2016; De Mendoza et al., 2020). These results indicate that photosynthetic function emerges from the interplay between excitation transport, regulation, dissipation, and environmental variability under natural illumination.

This Perspective discusses photosynthetic function in the context of dynamically regulated excitation flow under natural illumination. These processes operate under physical constraints imposed by finite excitationtransfer and charge-separation rates, thermal fluctuations, stochastic photon-arrival statistics, energetic disorder, and the need for controlled dissipation to prevent photodamage. Together, these constraints reflect the interplay between quantum dynamics, statistical fluctuations, and thermodynamic regulation in open biological systems (May and Kühn, 2011; Scholes et al., 2011, Scholes et al., 2017).

Within this framework, photosynthetic architectures can be interpreted as the result of evolutionary tuning not only to molecular constraints but also to the statistical properties of the light environment. This perspective emphasizes robust and adaptable energy flow under fluctuating environmental conditions rather than maximization of excitation-transfer efficiency alone.

## 2 Physics as a framework for understanding photosynthetic function

Photosynthesis is fundamentally a physical process involving light absorption, electronic excitation, charge separation, and the transport and dissipation of energy across molecular networks. Although biological function emerges from molecular structure, its operation is constrained by physical principles governing energy flow, dissipation, and regulation in nonequilibrium environments. While biological and chemical perspectives are essential for understanding molecular organization and function, physical descriptions provide the quantitative framework needed to analyze energy transport, environmental fluctuations, and multiscale dynamical regulation under natural illumination. Understanding stable operation under sunlight therefore requires quantitative descriptions capable of accounting for energy transfer, environmental interactions, and stochastic excitation dynamics under fluctuating conditions (Blankenship, 2014; Scholes et al., 2011).

Within pigment–protein complexes, excitation transfer arises from electronic coupling between nearby chromophores. Following photon absorption, electronic excitation may become transiently delocalized across multiple coupled pigments rather than remaining localized on a single chromophore, a phenomenon commonly associated with excitonic quantum coherence. These partially delocalized excitonic states enable energy propagation through interconnected antenna networks toward reaction centers via multiple transfer pathways (Förster, 1948; van Amerongen et al., 2000; Blankenship, 2014). Because pigment–protein complexes function as open physical systems interacting continuously with surrounding proteins, solvent motion, and thermal noise, excitation dynamics are strongly influenced by their molecular environment.

The protein scaffold organizing pigment arrangements therefore acts not merely as passive structural support but as an active physical environment that modulates coupling strengths, energetic landscapes, vibrational interactions, and dynamically fluctuating system–environment interactions (May and Kühn, 2011; van Grondelle and Novoderezhkin, 2006; Jumper et al., 2018; Ruan and Weng, 2026). From this modern perspective, excitation dynamics cannot be understood solely in terms of isolated electronic coherence, but instead emerge from the interplay between partially delocalized excitonic states, molecular vibrations, thermal fluctuations, and environmentally regulated dissipation. Vibronic dynamics arise when excitonic states interact resonantly with specific molecular vibrations, generating mixed excitonic– vibrational states shaped by environmental interactions. More recent spectroscopic studies further suggest that many experimentally observed long-lived oscillatory signatures originate predominantly from excitonic– vibrational interactions and environmentally modulated vibronic dynamics, rather than from sustained purely electronic coherence (Higgins et al., 2021; Policht et al., 2022). Environmental and vibrational interactions may therefore facilitate excitation redistribution, modulate resonant transfer pathways, prevent excessive localization, and stabilize robust energy transport across interconnected molecular networks operating under physiological conditions (Huelga and Plenio, 2013; Scholes et al., 2017, Scholes et al., 2011; Ruan and Weng, 2026). **Figures 1a, b** schematically illustrates excitation delocalization, vibronic interactions, environmentally regulated transport, and directed energy transfer toward reaction centers, together with the interplay between coherent dynamics, dissipation, and photochemical conversion.

From this perspective, photosynthesis can be understood as a regulated physical process emerging from interactions between molecular architecture and environmental dynamics. This framework motivates a closer examination of the experimental observations that first revealed quantum features of excitation transport in photosynthetic systems.

## 3 From early to modern quantum insights into photosynthetic energy transfer

Quantum coherence refers to the transient delocalization of electronic excitations across multiple coupled pigments while preserving phase relationships over finite timescales. In photosynthetic systems, this delocalization allows excitation energy to explore interconnected transfer pathways before relaxation and dissipation dominate. In ultrafast spectroscopy, these coherences appear as oscillatory signals or quantum beats on femtosecond-to-picosecond timescales. **Figures 1a, b** illustrates excitation delocalization, environmentally regulated transport, vibronic interactions, and directed energy transfer in photosynthetic antenna systems.

The development of ultrafast spectroscopic techniques, particularly two-dimensional electronic spectroscopy, enabled the observation of transient oscillatory signals in photosynthetic pigment–protein complexes (Engel et al., 2007; Panitchayangkoon et al., 2010; Collini et al., 2010). These pioneering experiments were initially interpreted as evidence for long-lived electronic coherence and wave-like excitation transport across photosynthetic networks, stimulating extensive work on the possible functional role of quantum coherence in photosynthetic energy transfer (Scholes et al., 2017). Although these experiments revealed genuine ultrafast quantum dynamics, later studies showed that spectroscopic observability alone does not imply a dominant physiological role for long-lived electronic coherence. Early theoretical descriptions emphasized wave-like excitation dynamics within structured molecular environments and proposed that partially coherent dynamics could enhance excitation redistribution. Subsequent theoretical and spectroscopic studies progressively showed that many long-lived oscillatory signatures could not be explained solely by persistent electronic coherence and instead involved important vibrational and vibronic contributions (Lim et al., 2015; Chin et al., 2013; Romero et al., 2014). More recent experimental and theoretical studies instead indicate that many long-lived oscillatory features detected in ultrafast spectroscopy are more consistently explained by environmentally influenced vibronic interactions and excitonic–vibrational coupling than by persistent purely electronic coherence (Arsenault et al., 2021; Policht et al., 2022; Jumper et al., 2018; Cao et al., 2020; Ruan and Weng, 2026).

Many pioneering ultrafast spectroscopic studies were performed on photosynthetic bacteria and algae, whose pigment–protein complexes constitute experimentally accessible and structurally well-characterized model systems (Engel et al., 2007; Panitchayangkoon et al., 2010; Collini et al., 2010). These studies stimulated broad interest in excitonic dynamics across diverse photosynthetic organisms, including cyanobacteria and higher plants, where excitation transport likewise occurs within densely coupled pigment–protein networks (Blankenship, 2014; van Amerongen et al., 2000; Croce and van Amerongen, 2014; van Amerongen and Croce, 2013).

It is important to distinguish between electronic, vibrational, and vibronic coherence. Electronic coherence corresponds to coherent superpositions between excitonic states and typically decays rapidly due to interactions with the surrounding thermal environment. Vibrational coherence instead arises from coherent nuclear motion and may persist longer than purely electronic coherence. Vibronic coherence emerges from resonant coupling between excitonic states and specific molecular vibrations, generating coupled excitonic–vibrational dynamics that can contribute to long-lived oscillatory behavior in photosynthetic complexes (Chin et al., 2013; Romero et al., 2014; Lim et al., 2015). More recent experimental and theoretical studies further indicate that many oscillatory signatures observed in ultrafast spectroscopic experiments are more consistently explained by environmentally influenced vibronic interactions than by persistent purely electronic coherence (Arsenault et al., 2021; Policht et al., 2022; Jumper et al., 2018; Cao et al., 2020; Ruan and Weng, 2026).

Conceptual models such as environment-assisted quantum transport (ENAQT) proposed that moderate environmental interactions could facilitate excitation redistribution by preventing excessive localization of excitonic states within pigment networks (Plenio and Huelga, 2008; Rebentrost et al., 2009b; Huelga and Plenio, 2013). Although these models provided important insight into environmentally assisted transport, more recent perspectives emphasize that the biological environment actively regulates excitation dynamics through vibrational coupling, structural fluctuations, and dissipation. In this modern interpretation, excitation transport emerges from the interplay between transient excitonic delocalization, vibronic interactions, environmental fluctuations, and regulated dissipation under natural illumination (Jumper et al., 2018; Cao et al., 2020; Arsenault et al., 2021; Higgins et al., 2021; Policht et al., 2022; Ruan and Weng, 2026; Jha et al., 2026).

Importantly, most early insights into coherent dynamics were obtained under coherent laser excitation rather than thermal sunlight. Natural photosynthesis operates under incoherent and fluctuating illumination, where excitation events are sparse, temporally irregular, and continuously influenced by environmental noise and regulatory processes. These considerations motivated subsequent efforts to understand how excitation transport, dissipation, and photoprotection operate collectively under realistic environmental conditions.

## 4 Quantum photosynthesis under natural illumination: physical constraints and timescales

Most experimental and theoretical studies of quantum coherence in photosynthesis have been performed under controlled laser excitation, where coherent optical pulses selectively probe ultrafast excitation dynamics. Natural photosynthesis, however, operates under sunlight –a broadband thermal radiation field fundamentally different from coherent laboratory sources. Thermal light exhibits stochastic intensity fluctuations, finite temporal correlations, and bursted photon arrival statistics arising from photon bunching (Mandel and Wolf, 1995).

This distinction between coherent laboratory excitation and natural thermal illumination has important implications for the physical interpretation of photosynthetic energy transport. In particular, previous theoretical work proposed that the temporal statistics of sunlight itself may play a central role in shaping excitation dynamics under physiological conditions (De Mendoza et al., 2020, De Mendoza et al., 2017). Those studies showed that thermal radiation generates sparse burst-like excitation events separated by relatively long intervals, producing dynamically fluctuating excitation regimes rather than continuous coherent driving. Because photon absorption events occur stochastically while reaction centers require finite reopening and processing times following charge separation, burst-like excitation delivery under sunlight can transiently exceed the instantaneous photochemical utilization capacity of the system. As a result, intermittent excitation accumulation, transient reaction-center saturation, enhanced dissipation, and fluctuating excitation losses may emerge even under moderate average illumination conditions (De Mendoza et al., 2020). Photosynthetic systems therefore operate within dynamically regulated regimes in which stochastic photon arrival statistics influence excitation transfer, dissipation, and photoprotection (Mančal and Valkunas, 2010; Fassioli et al., 2014; Brumer, 2018; De Mendoza et al., 2020). Under these conditions, excitation transport is shaped not only by pigment coupling and molecular structure, but also by fluctuating illumination conditions.

A defining feature of photosynthetic operation under natural illumination is the coexistence of processes spanning widely separated timescales. Ultrafast excitonic and vibronic dynamics occur on femtosecond to picosecond timescales (Engel et al., 2007; Panitchayangkoon et al., 2010; van Grondelle et al., 1994; van Grondelle and Novoderezhkin, 2006; Huelga and Plenio, 2013), whereas reaction-center turnover, electron transport, and photoprotective regulation occur on microsecond to millisecond timescales (Blankenship, 2014; Ruban, 2016). **Figure 1c** summarizes this multiscale organization, linking ultrafast excitation dynamics within antenna complexes to slower biochemical and regulatory processes.

Under sunlight, photon absorption events within individual antenna systems are typically sparse and temporally irregular, with burst-like fluctuations separated by microsecond or longer intervals (Mančal and Valkunas, 2010; Fassioli et al., 2014; De Mendoza et al., 2020). Excitation events are therefore generally isolated relative to internal relaxation and dissipation timescales, such that biological function is governed primarily by dynamically regulated energy flow rather than by continuously sustained coherent evolution.

Environmental fluctuations also play important roles under natural illumination. As described previously, molecular vibrations, thermal noise, and structural fluctuations continuously influence excitation dynamics in biological environments, contributing to decoherence, relaxation, dissipation, and excitation redistribution (Breuer and Petruccione, 2002; Huelga and Plenio, 2013). Moderate environmental interactions may therefore regulate excitation flow, facilitate excitation redistribution, prevent excessive localization, and stabilize robust energy transport across coupled pigment networks rather than acting solely as sources of disorder.

Photosynthetic function therefore emerges from the interplay between quantum excitation dynamics, stochastic photon absorption, environmental fluctuations, and slower regulatory mechanisms operating across multiple temporal scales (Brumer, 2018; Mančal and Valkunas, 2010; Fassioli et al., 2014).

## 5 Functional implications: robustness and photoprotection

Natural illumination imposes functional constraints on photosynthetic systems because excitation delivery fluctuates across multiple temporal and environmental scales. Although excitation events are typically sparse, transient increases in excitation density may temporarily exceed the system’s capacity for photochemical utilization, increasing the risk of excitation accumulation and photodamage (Blankenship, 2014; Ruban, 2016). Photosynthetic systems therefore operate as regulated networks in which excitation energy is continuously redistributed and dissipated to maintain stable operation under fluctuating conditions. Theoretical studies of open photosynthetic systems under thermal-light excitation further show that stochastic excitation delivery and finite reaction-center processing times strongly influence excitation accumulation and dissipation dynamics (Scholes et al., 2017; De Mendoza et al., 2020).

Different photoprotective mechanisms help prevent photodamage by limiting the accumulation of excess excitation energy under high illumination conditions (Ruban, 2016; Demmig-Adams and Adams, 2006; Niyogi, 1999; Müller et al., 2001; Horton et al., 1996). Under excess light, overexcited chlorophyll molecules may populate long-lived triplet states that can transfer energy to molecular oxygen, generating highly reactive singlet oxygen species capable of damaging photosynthetic membranes, antenna complexes, and reaction centers (Blankenship, 2014; Ruban, 2016). Carotenoid-mediated triplet quenching constitutes an important constitutive photoprotective mechanism that operates continuously by rapidly accepting triplet excitation energy from chlorophylls and dissipating it non-radiatively before reactive oxygen species can form. In contrast, non-photochemical quenching (NPQ) corresponds to a regulated and dynamically activated photoprotective response that is induced under sustained excess illumination conditions (Ruban, 2016; Demmig-Adams and Adams, 2006; Niyogi, 1999; Müller et al., 2001; Horton et al., 1996). In plants and algae, NPQ involves pH-dependent activation of antenna proteins, xanthophyll-cycle pigments, and structural reorganization of light-harvesting complexes that redirect excitation energy away from photochemical conversion toward controlled thermal dissipation.

Recent spectroscopic and theoretical studies further suggest that relatively small structural and vibrational changes within antenna complexes may dynamically regulate the balance between light harvesting and dissipation by modulating excitonic couplings and energy-transfer pathways under fluctuating illumination conditions (Ruan and Weng, 2026; Romero et al., 2014; Jha et al., 2026; Higgins et al., 2021).

In contrast, dissipative behavior may also emerge intrinsically from the dynamical organization of photosynthetic systems, even in the absence of dedicated photoprotective activation. Under fluctuating excitation conditions, finite reaction-center processing times can transiently limit excitation utilization, enhancing excitation losses and dissipation through purely dynamical bottlenecks (De Mendoza et al., 2020). Photosynthetic function therefore depends not only on efficient excitation transfer, but also on maintaining a regulated balance between energy delivery, utilization, and dissipation (Blankenship, 2014; Rochaix, 2014).

**Figure 2:**
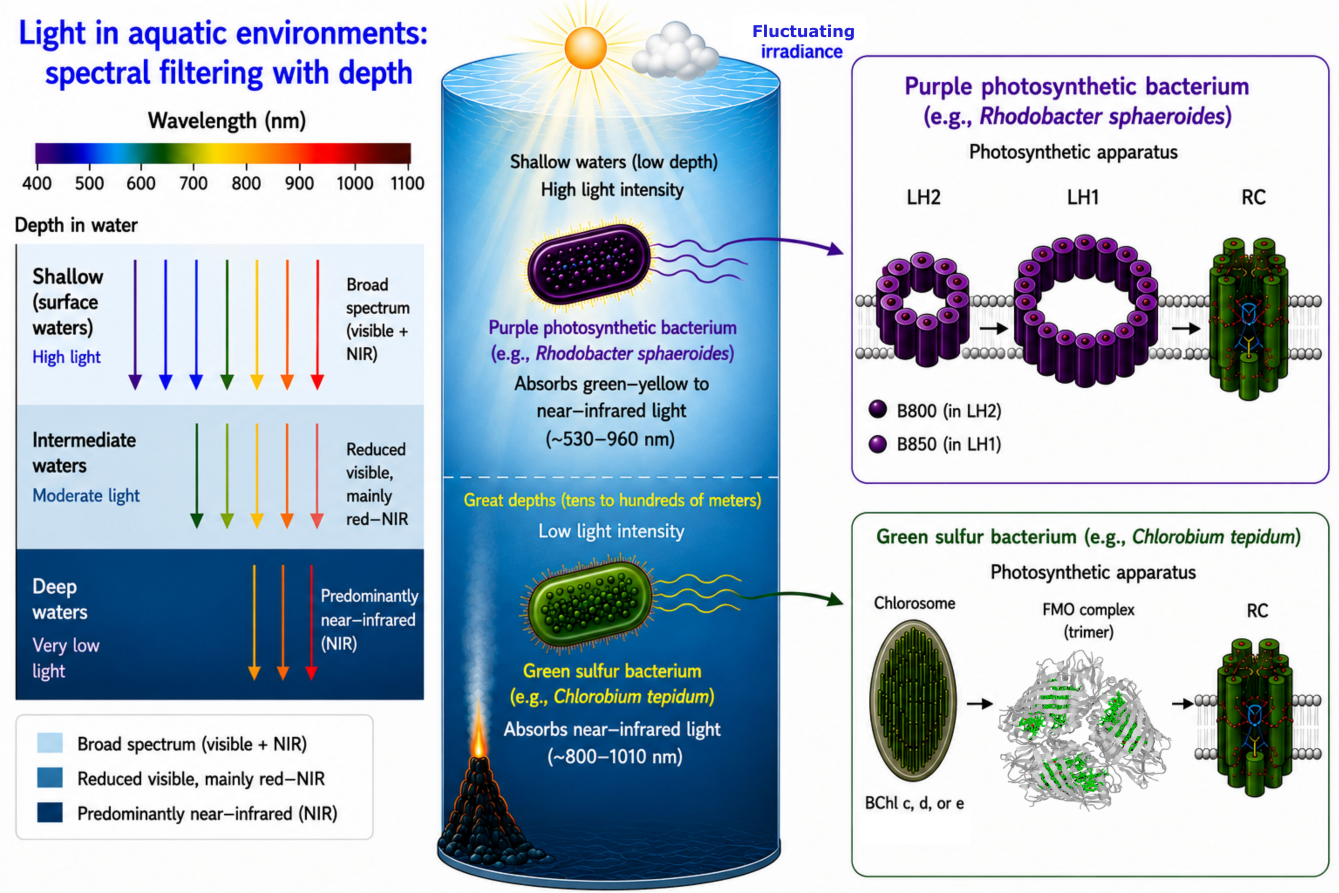
Photosynthetic adaptation to distinct aquatic light environments. Light availability in aquatic environments changes with depth due to spectral filtering, scattering, attenuation, and fluctuating irradiance conditions. Shallow waters receive broad-spectrum visible and near-infrared (NIR) illumination, whereas deeper environments operate under reduced and spectrally modified light conditions. Purple photosynthetic bacteria (e.g., *Rhodobacter sphaeroides*) typically inhabit shallow waters and employ membrane-embedded LH2/LH1 antenna complexes surrounding reaction centers (RCs), while green sulfur bacteria (e.g., *Chlorobium tepidum*) inhabit low-light environments and utilize large chlorosomes coupled to Fenna–Matthews–Olson (FMO) complexes. These architectures illustrate how photosynthetic systems adapt to distinct environmental constraints through regulated excitation transport, dissipation, and light harvesting under variable illumination conditions rather than through a single optimized transfer mechanism. Structural representations are based on experimentally resolved bacterial antenna and FMO architectures reported in the literature (Blankenship, 2014; van Amerongen et al., 2000; Croce and van Amerongen, 2014; Engel et al., 2007). The conceptual interpretation emphasizes environmentally regulated excitation transport and vibronic interactions under natural illumination conditions (Cao et al., 2020; Ruan and Weng, 2026).

Experimental and theoretical studies across diverse ecological and physiological contexts indicate that photosynthetic organisms continuously regulate excitation transport, dissipation, antenna organization, and photoprotective responses in order to maintain stable photosynthetic function under fluctuating environmental illumination rather than under a single optimized operating regime (Kromdijk et al., 2016; Niyogi and Truong, 2013; Kirilovsky, 2015; Rochaix, 2014; Johnson, 2016; Higgins et al., 2021). **Figure 2** illustrates how photosynthetic bacteria exhibit structural and light-harvesting adaptations to distinct light environments across aquatic depth gradients. Purple photosynthetic bacteria adapted to shallow environments typically operate under higher irradiance conditions using membrane-embedded antenna complexes, whereas green sulfur bacteria inhabiting deeper low-light environments rely on chlorosomes and specialized transfer complexes adapted for weak near-infrared illumination. These structural differences illustrate how photosynthetic architectures reflect adaptation to the spectral composition, intensity, and variability of natural light environments under distinct physical constraints.

These observations support the view that photosynthesis operates as an energy-regulated physical system shaped by evolutionary pressures favoring stability and adaptability under natural illumination. Rather than maximizing a single performance metric such as transfer efficiency, photosynthetic systems operate within regimes that prioritize stable and safe functionality across fluctuating environmental conditions.

## 6 Discussion: future directions in quantum photosynthesis

The study of quantum effects in photosynthesis has evolved from the early observation of coherent ultrafast dynamics under controlled laboratory conditions toward a more modern view in which excitation transport under physiological conditions is regulated by vibronic interactions, environmental fluctuations, and dissipation under natural illumination (Cao et al., 2020; Jumper et al., 2018; Ruan and Weng, 2026; Jha et al., 2026; Harush and Dubi, 2021). This shift has highlighted the importance of moving beyond idealized experimental settings toward theoretical and experimental approaches that incorporate realistic illumination conditions, including continuous excitation, fluctuating spectra, thermal noise, and burst-like photon statistics associated with thermal radiation fields. Experimental studies performed under controlled sunlight-like conditions, and across diverse photosynthetic organisms, will therefore be essential for testing predictions derived from models of environmentally regulated excitation dynamics.

Several open questions directly connect physical mechanisms with biological function: What determines the excitation density at which photoprotective responses are triggered? How do burst-like photon statistics and environmental fluctuations affect the robustness of excitation redistribution within antenna networks? To what extent do spectral variations in natural light environments shape the structural organization of pigment–protein complexes across photosynthetic species? Addressing these questions will require integrating quantitative physical models with experimental observations across multiple biological scales, including mechanisms governing excitation regulation under natural illumination such as non-photochemical quenching, reaction-center turnover, and controlled dissipation.

A particularly important frontier concerns the physical role of reaction centers under natural illumination. While the structure and primary charge-separation mechanisms of reaction centers are well characterized, their functional behavior within dynamically regulated excitation networks operating under stochastic sunlight remains incompletely understood (Blankenship, 2014; Scholes et al., 2011). In natural environments, reaction centers act not only as sinks for excitation but also as dynamic nodes whose turnover dynamics, saturation behavior, and recovery times influence excitation accumulation, dissipation, and overall system stability across multiple timescales (Ruban, 2016; Blankenship, 2014). Recent theoretical work further suggests that interactions between excitation dynamics and the temporal statistics of thermal radiation can influence how excitation accumulates and dissipates under continuous illumination (Mančal and Valkunas, 2010; Brumer, 2018; De Mendoza et al., 2020; Manrique et al., 2016). These interactions may generate transient imbalances favoring photoprotective activation or excitation losses associated with finite reactioncenter processing times. Extending this line of investigation across different photosynthetic organisms and environmental regimes will be essential for identifying general physical constraints governing energy regulation and photoprotection.

Understanding regulated excitation dynamics under realistic illumination also raises broader questions regarding adaptation across ecological and evolutionary contexts. Photosynthetic organisms occupying distinct ecological niches experience markedly different spectral and irradiance conditions, suggesting that photosynthetic architectures evolve under environment-specific physical constraints (Kirilovsky, 2015; Niyogi and Truong, 2013; Kromdijk et al., 2016; Manrique et al., 2015). Comparative studies across photosynthetic lineages—including anoxygenic bacteria, cyanobacteria, algae, and higher plants—supported by physically grounded models, may help identify general principles governing adaptability and resilience under changing illumination regimes.

Insights gained from understanding these biological mechanisms may, in turn, inform the design of artificial light-harvesting technologies capable of operating under realistic environmental conditions. Rather than optimizing solely for peak efficiency under idealized settings, future technological strategies may benefit from design principles that prioritize robustness, adaptability, and controlled energy dissipation. In this way, photosynthetic systems serve not only as biological models but also as physical platforms for developing design strategies inspired by experimentally supported physical principles.

These directions illustrate how integrating physical principles with biological insight can enable integrated understanding of photosynthetic function under realistic environmental conditions. Continued integration of experimental observations, theoretical modeling, and ecological perspectives will be essential for linking molecular-scale excitation dynamics to organism-level function and evolutionary adaptation across fluctuating environments. More broadly, these perspectives may help establish general physical principles governing regulated energy flow, adaptation, and robust energy conversion in complex living systems operating under fluctuating environmental conditions.

## Data Availability

The original contributions presented in the study are included in the article/supplementary material. Further inquiries can be directed to the corresponding author.
